# Adventitious Error and Its Implications for Testing Relations Between Variables and for Composite Measurement Outcomes

**DOI:** 10.1007/s11336-024-09980-7

**Published:** 2024-07-04

**Authors:** Paul De Boeck, Michael L. DeKay, Jolynn Pek

**Affiliations:** Department of Psychology, The Ohio State University, 1827 Neil Avenue, Columbus, OH 43210 USA

**Keywords:** adventitious error, covariance matrices, inferential uncertainty, heterogeneity of effects, power, measurement uncertainty

## Abstract

Wu and Browne (Psychometrika 80(3):571–600, 2015. https://doi.org/10.1007/s11336-015-9451-3; henceforth W &B) introduced the notion of adventitious error to explicitly take into account approximate goodness of fit of covariance structure models (CSMs). Adventitious error supposes that observed covariance matrices are not directly sampled from a theoretical population covariance matrix but from an operational population covariance matrix. This operational matrix is randomly distorted from the theoretical matrix due to differences in study implementations. W &B showed how adventitious error is linked to the root mean square error of approximation (RMSEA) and how the standard errors (SEs) of parameter estimates are augmented. Our contribution is to consider adventitious error as a general phenomenon and to illustrate its consequences. Using simulations, we illustrate that its impact on SEs can be generalized to pairwise relations between variables beyond the CSM context. Using derivations, we conjecture that heterogeneity of effect sizes across studies and overestimation of statistical power can both be interpreted as stemming from adventitious error. We also show that adventitious error, if it occurs, has an impact on the uncertainty of composite measurement outcomes such as factor scores and summed scores. The results of a simulation study show that the impact on measurement uncertainty is rather small although larger for factor scores than for summed scores. Adventitious error is an assumption about the data generating mechanism; the notion offers a statistical framework for understanding a broad range of phenomena, including approximate fit, varying research findings, heterogeneity of effects, and overestimates of power.

A common problem of estimating a covariance structure model (CSM; e.g., a factor model or latent growth curve model) is that the estimated model is usually somewhat misspecified (e.g., see MacCallum, 2003). The specified model is at best only an approximate model of the data, which is an exemplification of Box’s (1976) statement that all models are wrong (but some are useful). Goodness-of-fit (GOF) indices have been developed to evaluate how well the estimated CSM fits the data. For example, the root mean squared error of approximation (RMSEA) is a popular GOF index, and it is common practice to accept an estimated model as sufficiently approximate if certain cutoff values of GOF indices are met (e.g., $\textrm{RMSEA}\le 0.05$; Browne & Cudeck, 1993). One reason for a model being approximate is that small sources of covariance, which could be treated as minor factors or error covariances, are omitted from the specified model and treated as nuisance instead (e.g., Tucker et al., 1969). This practice can lead the model to be rejected based on statistical criteria such as GOF $\chi ^{2}$ test.

There are two different approaches to deal with imperfect model fit. One approach is model respecification, for example, following suggestions based on modification indices (Sörbom, 1989), which may result in model extensions that no longer lead to a statistical rejection of perfect model fit. However, potential issues are the power of the GOF test and the possible ad hoc nature and non-replication of model modifications (e.g., MacCallum, 1986). An alternative approach, which we follow here, is to accept the approximate nature of models (Browne and Cudeck, 1993) while allowing that models can sometimes have a perfect fit (e.g., Bollen, 1989; Jöreskog, 1969). Although the approximate nature of models and the consequent notion of approximate fit are broadly accepted (e.g., using the RMSEA), the discrepancy between the theoretical and approximate models has received limited statistical formalization (but see Cudeck & Henly, 1991).

Wu and Browne (2015; henceforth W &B) proposed the concept of *adventitious error* within a statistical framework for formalizing the approximate nature of CSMs instead of either simply ignoring or explicitly accounting for minor sources of covariance beyond sampling variation (i.e., specifying a more complex model). W &B formalized adventitious error as a random distortion of the “theoretical population” covariance matrix due to the specificities of each implemented study but unrelated to sampling variation, yielding a different “operational population” covariance matrix for each study. Sampling variation for each study is centered at the operational population covariance matrix and not at the theoretical population covariance matrix. In short, adventitious error and sampling variation are separate, additive sources of uncertainty. If approximate fit stems from adventitious error—a random source—rather than a systematic source, it does not follow that the model parameters are biased: “Under the assumption of large sample size and small adventitious error, the estimators for covariance structure parameters are consistent and asymptotically normally distributed” (W &B, p. 595).

Embracing adventitious error requires the analyst to abandon the ideas of a fixed reality and fixed population models. Fixed population models are not sufficiently flexible to take into account the undeniable complexity of realities that interest psychologists and other social scientists. To model the complexity explicitly and fully, the models would need to be as complex as the phenomena being modeled. A common but necessarily incomplete approach to explaining deviations from a fixed population model (i.e., beyond sampling variation) is to include moderators as explanatory variables, so that variations in one or more effects might be attributed to different levels of one or more moderators. Such patterns can make interpretation difficult and raise concerns about replicability.

The random nature of adventitious error allows for a different approach. Adventitious error does not provide specific conceptual explanations for deviations from a fixed population model because randomness does not qualify as an explanation. Instead, adventitious error provides a statistical framework that quantifies the approximate nature of models, resulting in downstream adjustments to inferential devices, including more conservative significance tests and wider confidence intervals. We argue that, in contrast, fixed population models overestimate the certainty in such inferences (i.e., that fixed population models are misspecified).

There are precedents for a randomness approach. For example, the scientific community has been willing to accept that higher-order interactions are absorbed in a random term in ANOVA (e.g., McGraw & Wong, 1996), to accept random-effect models in meta-analysis (e.g., Borenstein et al., 2009), and to approach large-scale replication projects with random-effect modeling (e.g., McShane et al., 2022). Perhaps the most important precedent is the concept of measurement error, which is so well established that one would not even think of trying to describe every specific source of error. Instead, we treat random deviations due to measurement error as if the term “measurement error” is self-explanatory.

Adventitious error is distinct from measurement error because adventitious error refers to the covariance among variables in a study. Although conceptually distinct, adventitious error and measurement error share the same feature of randomness, and, as will be explained, adventitious error has consequences for composite measurement outcomes as well. Just as measurement error refers to a specific combination of influences and co-occurrences associated with an individual measurement outcome, adventitious error refers to a specific combination of influences and co-occurrences associated with the covariance of variables in an individual study. Although different from measurement error, the effects of adventitious error can trickle down to composite measurement outcomes, as will be shown.

## Overview

Our contribution here is to discuss and illustrate possible implications of adventitious error in a context broader than the CSM framework. For example, descriptive correlations between variables in the absence of an explicit model for the correlations should also be subject to adventitious error. There is no reason why adventitious error should occur only when variables are expressed within in a CSM model or why adventitious error should not also be relevant for the uncertainty of relations between variables in a more general sense. We may expect that distortions to variance-covariance values among manifest variables (MVs), due to the specificities of individual studies, are independent of the type of analysis that follows the data collection. Thus, the occurrence of adventitious error does not depend on the use of a CSM and is not necessarily reflected in the GOF of the model. For example, adventitious error may lead to varying relations between a pair of variables (e.g., varying correlations) based on the specificities of studies, but it would not lead to GOF issues in a simple regression model because the model is saturated (i.e., GOF will be perfect). Stated differently, the estimated regression effect may deviate from the true effect due to adventitious error even when the model perfectly fits the data. We accept that random distortions of covariances among MVs are independent of the data-analytic framework applied to the data. The CSM context is a special case in which adventitious error is reflected in GOF if the operational population covariance matrix deviates from the theoretical population covariance matrix in a way that is described in Sect. 1.1. Importantly, W &B also showed that the RMSEA GOF index gives us an indication of the extent of adventitious error, as we explain in the same section.

Our further contribution consists of three parts. In Part 1, we introduce a *formalization of adventitious error and its impact on uncertainty*, which is based on the work of W &B on CSMs. In Part 2, we illustrate the consequences of accepting adventitious error for the* relations between variables*, regardless of whether these relations are modeled within the CSM framework. Three different sections of Part 2 are devoted to illustrations of the consequences, with each section designed to answer a different question:

Question 1: Are the inferential consequences of adventitious error in a CSM context generalizable beyond the CSM context, such as to simple relations between MVs?

Question 2: How might one interpret the heterogeneity of effects such as correlations in meta-analysis from the perspective of adventitious error?

Question 3: What are the consequences of adventitious error for statistical power for testing the significance of correlations and regression slopes in simple regression?

These three sections provide only simple illustrations to give the reader an initial idea of the possible consequences of adventitious error.

Finally, in Part 3, we discuss a way to estimate the impact of adventitious error on the *uncertainty of composite measurement outcomes* (e.g., summed scores and factor scores). The uncertainty of measurement outcomes depends on their variability. A well-known source of variability is measurement error, formulated as deviations from the expected value of measurement outcomes in a standard condition. We believe that adventitious error also contributes to measurement uncertainty, although in an additional way compared with the common measurement error notion.

As a precautionary note, we want to clarify our claims. First, although it would follow from adventitious error that CSMs are approximate and that minor nuisance sources of covariance occur, these latter phenomena do not prove the occurrence of adventitious error. However, we do claim that phenomena other than those that led to the notion of adventitious error, such as the heterogeneity of effects and the overestimation of statistical power can also be explained as stemming from adventitious error. Second, the adventitious error rate cannot be easily estimated. Although W &B derived a way to connect the RMSEA GOF index to the adventitious error rate in the context of CSMs, we do not know of any other model or analysis for which the adventitious error rate is mathematically related to a statistic derived from the analysis. Furthermore, we recognize that most empirical studies do not make use of CSMs. For all these reasons, we utilize a benchmark rate of adventitious error to illustrate its effects, as explained in Sect. 1.2.

## Formalization of Adventitious Error

### Sampling Error and Adventitious Error for CSMs

The common statistical approach for CSMs is to assume multivariate normality for data associated with a $p\times p$ population covariance matrix $\varvec{\varSigma }$, with *p* as the number of MVs. A $p\times p$ observed covariance matrix ${\varvec{S}}$ is sampled from a Wishart distribution with $\varvec{\varSigma }/n$ as its scale matrix and *n* (sample size *N* minus 1) as its degrees of freedom: ${\varvec{S}}\,\sim W(\varvec{\varSigma }/n,n)$. Stated differently, the sampling distribution for ${\varvec{S}}$ is centered at $\varvec{\varSigma }$ with dispersion 1/*n*. Increasing *n* decreases sampling error.

**Fig. 1:**
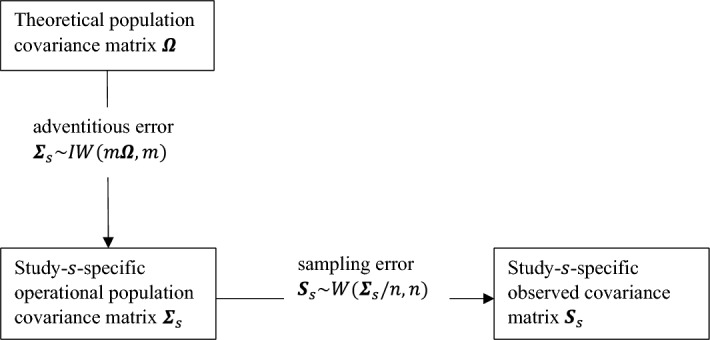
Sampling scheme for an operational population covariance matrix and an observed covariance matrix. n = sample size N - 1 and m is the degrees of freedom of an inverse Wishart distribution.

Figure 1 gives a conceptual representation of how the observed covariance matrix is generated in the presence of adventitious error. A theoretical population model covariance matrix $\varvec{\varOmega }$ is first randomly distorted in each study, indexed by *s*, so that the operational population covariance matrix $\varvec{\varSigma }_{s}$ deviates from the theoretical population covariance matrix $\varvec{\varOmega }$ due to the specificities of each study (i.e., how each study is uniquely implemented). Next, the observed covariance matrix ${\varvec{S}}_{{\varvec{s}}}$ is sampled from $\varvec{\varSigma }_{s}$. The random distortion of the theoretical $p\times p$ covariance matrix $\varvec{\varOmega }$ is based on an inverse Wishart distribution: $\varvec{\varSigma }_{s} {\sim } IW(m\varvec{\varOmega },m),$ with $m\varvec{\varOmega }$ as the scale matrix and *m* as its degrees of freedom. The adventitious error distribution is centered at $\varvec{\varOmega }$, with dispersion 1/*m*. Thus, the operational covariance matrix $\varvec{\varSigma }_{s}$ is randomly perturbed from $\varvec{\varOmega }$, and the observed covariance matrix ${\varvec{S}}_{s}$ is then sampled from $\varvec{\varSigma }_{s}$ and not from $\varvec{\varOmega }$.

*Notes*. (1) The expected value of the IW distribution centered at $\varvec{\varOmega }$ is not $\varvec{\varOmega }$, but $m\varvec{\varOmega }/(m-p-1)$ instead. (2) Although the IW distribution is used within a Bayesian framework as a conjugate prior for covariance matrices, the distribution as such also makes sense in a frequentist perspective. (3) Adventitious error makes the uncertainty about parameter estimates aleatory. Aleatory uncertainty about a parameter estimate cannot be reduced with more information (see O’Hagan, 2004, for more details). Specifically, the variability of relations between variables cannot be reduced by more information, such as when multiple studies are conducted.

### Standard Errors in the Presence of Adventitious Error

W &B derived a crucial link between GOF and uncertainty due to adventitious error (if it occurs) within the context of CSM. The RMSEA is related to the dispersion of the IW distribution with scale matrix $\varvec{\varOmega }$:

1 $$\begin{aligned} \text {RMSEA}^{2}=1/m. \end{aligned}$$

The RMSEA has been developed to quantify imperfect model fit. An RMSEA of 0.00 indicates perfect fit, values of 0.05 or lower indicate good fit, and values higher than 0.05 up to 0.08 indicate satisfactory fit. Values beyond 0.08 suggest poor GOF (see Browne & Cudeck, 1993; Hu & Bentler, 1999). These benchmark values are rather arbitrary and may differ depending on the authors. For example, Hu & Bentler used 0.06 instead of 0.05 as a cutoff value. Although a CSM may be satisfactory in terms of GOF in the presence of adventitious error, there are implications for the uncertainty of inferences regarding CSM parameters (e.g., structural parameters, correlations among factors). It follows from W &B (p. 590) that in the presence of adventitious error, the standard errors (SEs) of parameter estimates increase by a factor

2 $$\begin{aligned} q=\sqrt{(1/m+1 /n)/(1/n)} \end{aligned}$$

so that the new SE is the traditional $\text {SE}\times q$. The first term within parentheses under the square root in Eq. (2), 1/*m*, is the extra source of variation (and thus uncertainty) in parameter estimates. Hereafter, SEs based on Eq. (2) are called *augmented* SEs. Recall that for CSMs, the dispersion of the IW distribution corresponds with RMSE$\textrm{A}^{\textrm{2}}$; see Eq. (1).

Whether or not a measurement model is estimated, it is idealistic to assume that the MVs used in a study have an observed covariance structure that is directly sampled from the theoretical population model $\varvec{\varOmega }$. A more realistic assumption is that in each study, an operational population model applies, so that an operational population covariance matrix $\varvec{\varSigma }_{s}$ underlies the covariances among the observed MVs.

To the extent that an $\textrm{RMSEA}\le 0.08$ is considered acceptable, one can use the 0.08 value to determine a benchmark value of the adventitious error rate when no RMSEA value is available or when one moves out of the CSM framework, such as for a simple regression analysis. Although somewhat arbitrary, in what follows, the benchmark value of 0.08 is used to illustrate the consequences of adventitious error. Based on Eq. (1), the dispersion of the IW distribution that would lead to an RMSEA of 0.08 is $1 / m=0.0064$. The corresponding degrees of freedom are approximately $1/0.0064=156$, which is to say that the uncertainty associated with a dispersion value of 0.0064 is comparable to the sampling error resulting from a sample size of approximately 157.

To illustrate the size of distortions that can result from this benchmark dispersion value, we conducted a small simulation study for a $4\times 4$ theoretical covariance matrix ($\varvec{\varOmega })$ with 1.00 on the diagonal and 0.49 in the off-diagonal cells (as for the MVs in Sect. 2.1) and with an *IW* dispersion of $1/m = 0.0064$. Based on 1000 replications, the average absolute deviation from the theoretical covariance matrix across all cells (including the diagonal cells) was 0.079. For nearly all illustrations in Parts 2 and 3, we use a dispersion of 0.0064. Occasionally, we also use an *IW* dispersion of 0.0025, corresponding to an RMSEA of 0.05 for CSMs. We do not intend to imply that in the absence of an RMSEA, a benchmark adventitious error rate should be adopted for inferences. Instead, we are interested in benchmark adventitious error rates only for illustrative purposes.

## Adventitious Error and Relations Between Variables

### Standard Errors for CSM and Linear Regression Parameter Estimates

In this section, we use small simulation studies to investigate whether the inflation factor *q* from Eq. (2) for the SEs of parameter estimates in the presence of adventitious error applies to CSMs (as already verified by W &B) and to other types of analysis such as simple linear regression. W &B derived the factor *q* for CSMs based on statistical theory to quantify the augmented uncertainty due to adventitious error (i.e., $\text {SE}\times q)$. Therefore, we call factor *q* from Eq. (2) the *theoretical augmented uncertainty factor* (TAUF). In addition, we generate data subject to adventitious error with the benchmark IW dispersion ($1/m = 0.0064$). Doing so allows us to obtain a *simulation-based augmented uncertainty factor* (SAUF), not derived from Eq. (2) but estimated from simulated data dividing the simulation-based SE with adventitious error by the simulation-based SE without adventitious error. SAUF, $\hat{q}$, is a simulation-based estimate of the TAUF, *q*. The purpose of the simulations is to answer Question 1 of Part 2: “Are the inferential consequences of adventitious error in a CSM context generalizable beyond the CSM context?”

We compare SAUF and TAUF for a CSM and for two simple linear regression models. For the CSM, we expect SAUF to correspond closely to TAUF, in line with the findings by W &B. For the simple linear regressions, the comparison of SAUF and TAUF gives us an indication of how well the TAUF for the benchmark adventitious error rate applies beyond the CSM context. These small simulations serve as a proof of concept, providing illustrations that might help to familiarize the reader with the augmented uncertainty due to adventitious error.

**Fig. 2:**
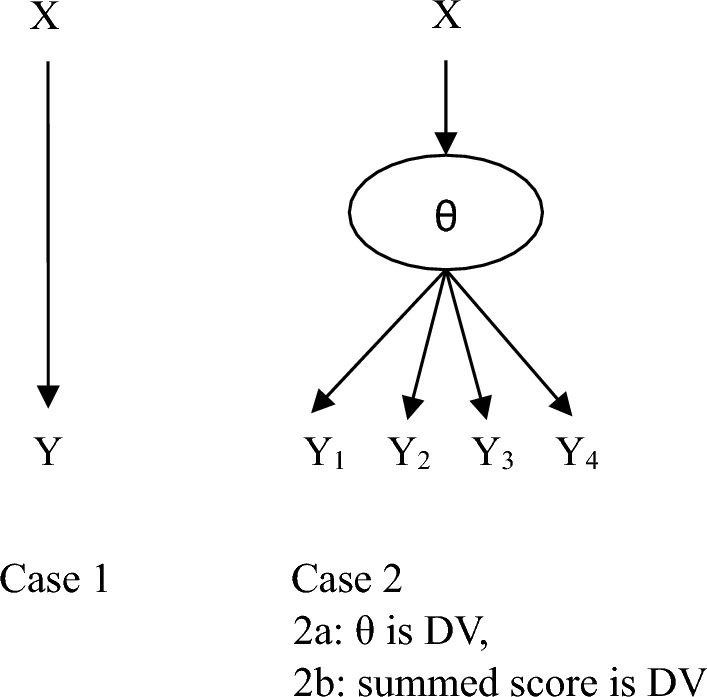
Cases 1, 2a and 2b of relations between variables.

More specifically, we consider three cases: 1, 2a, and 2b, as depicted in Fig. 2. Case 1 concerns two MVs, an independent variable (IV), *X*, and a dependent variable (DV), *Y*, using simple linear regression to estimate the effect of the IV. Case 2a concerns a CSM with a manifest IV, *X*,  and a latent DV, $\theta$, with four indicator variables, $Y_{1}$ to $Y_{4}$. Case 2b is inspired by case 2a in that the summed score of $Y_{1}$ to $Y_{4}$ is used instead of $\theta$ as a DV, again using simple linear regression to estimate the effect of the IV on the DV.

For each of the three cases, a covariance matrix $\varvec{\varOmega }$ is specified based on the following assumptions (which should not and—based on some additional simulations—do not appear to affect the main lines of the results):

- A variance of 1.00 is assigned to all variables in $\varvec{\varOmega }$, making $\varvec{\varOmega }$ a correlation matrix.
- The covariance of *X* with *Y* (case 1) is 0.25 and the covariances of *X* with $Y_{1}$ to $Y_{4}$ (cases 2a and 2b) are 0.35.
- The loadings for all four indicator variables (cases 2a and 2b) are 0.70, yielding covariances of 0.49.

The above choices lead to a $2 \times 2$ covariance matrix for case 1 and a $5\times 5$ covariance matrix for cases 2a and 2b. The simulations consist of four steps, with two levels of sample size ($N=200$ and 1000):

Step 1. Randomly distort $\varvec{\varOmega }$ 2000 times based on an IW dispersion parameter of $1/m =0.0064$, using the riwish() function from the MCMCpack version 1.4-5 package in R (Martin et al., 2011): riwish(*m*, omega $*\,m)$, where *m* is the inverse of the dispersion parameter 1/*m* and omega is $\varvec{\varOmega }$. This yields 2000 $\varvec{\varSigma }_{s}$ matrices for case 1 and 2000 $\varvec{\varSigma }_{s}$ matrices for case 2a and for case 2b. We report that using $1 /{(m-p+1)}$ instead of 1/*m* as the dispersion parameter derived from the RMSEA (as suggested by W &B) leads to nearly identical simulation results. Step 1 refers to the first arrow of adding adventitious error to ${\varvec{\Omega }}$ in Fig. 1 to obtain $\varvec{\varSigma }_{s}$ for $s =1,\ldots ,2000$.

Step 2. Generate data for two variables for case 1 and five variables for cases 2a and 2b, for each sample size (200 or 1000). Here, we use the mvrnorm( ) function (with empirical = FALSE) from the MASS package in R (Venables & Ripley, 2002), using the 2000 $\varvec{\varSigma }_{s}$ matrices and with means for the two variables generated based on a normal distribution with zero mean vector and $\text {SD} = 0.5$. The means and SDs are arbitrary values because the mean does not affect the covariances (under multivariate normality) and should therefore not affect the outcome. Step 2 refers to data generation based on $\varvec{\varSigma }_{s}$ as the operational covariance matrix. In Fig. 1, ${\varvec{S}}_{s}$ for $s = 1,\ldots , 2000$ is the covariance matrix of the generated data and is subject to sampling error.

Step 3. Given the generated variables for case 1 and each sample size, conduct a simple linear regression analysis for each of the 2000 generated data sets. Given the generated variables for cases 2a and each sample size, fit a structural equation model to each data set using the cfa( ) function from the lavaan version 0.6-5 package in R (Rosseel, 2012) using ML estimation. For case 2b, conduct a simple linear regression with the sum of $Y_{1}$ to $Y_{4}$ as the DV for each of the 2000 data sets. For all three analyses (cases 1, 2a, and 2b), the SD of the 2000 estimated effects is a simulation-based estimate of the augmented SE (i.e., $\text {SE}\times q)$ that serves as the numerator in the expression for SAUF. In sum, Step 3 provides us with a simulation-based estimate of the augmented SE with and without the use of a CSM (case 2a versus cases 1 and 2b, respectively).

Step 4. Repeat Step 2 for cases 1, 2a, and 2b, based on the corresponding $\varvec{\varOmega }$ matrix instead of the $\varvec{\varSigma }_{s}$ matrices; in other words, generate sample covariance matrices without distorting $\varvec{\varOmega }$ (i.e., omitting adventitious error). Given these sample covariance matrices, conduct the same analyses as in Step 3. For each of the three cases, determine the SD of the effect across the 2000 analyses. These SDs are simulation-based estimates of the traditional SE, serving as the denominator of the SAUF (i.e., of $\hat{q})$. We also used two alternative methods to generate the traditional SE estimates: (a) the median SE across the 2000 analyses from Step 3 and (b) the median SE across the corresponding 2000 analyses from Step 4. Because these two alternative approaches yield very similar SE estimates that are also very similar to the SD-based estimates from Step 4, we only report the latter.

Based on Steps 3 and 4, we have the necessary information to calculate the SAUF in each case, to be compared with the TAUF for a dispersion parameter of 0.0064. The results appear in Table 1. The simulation-based factors (SAUFs) corroborate the theoretical factors (TAUFs) based on Eq. (2), which are 1.51 and 2.72 for $N= 200$ and $N = 1000$, respectively. The larger ratios for $N = 1000$ may seem counterintuitive, but the traditional SE shrinks with sample size, whereas the 1/*m* term in the *q* factor in Eq. (2) does not shrink with sample size.

The results are slightly different depending on the case (1, 2a, or 2b) and the sample size ($N = 200$ or 1000). The SAUF is slightly larger for case 2a compared to the other two cases, at least for the larger sample size, and it is also slightly larger than the TAUF. More extensive simulations would be required to find out whether this observation replicates across other conditions.

The finding that the SAUF is a good approximation of the TAUF means that one can use TAUF to indicate how much the SE needs to be augmented, conditional on the sample size and the chosen adventitious error rate, not only for CSM parameter estimates but also for linear regression. We would expect this result to hold for adventitious error rates other than the benchmark rate used in the simulations.

**Table 1:** Theoretical and simulation-based augmented uncertainty factors for SEs.

	$N = 200$	$N = 1000$
TAUF	1.51	2.72
SAUF		
Case 1: $X \rightarrow Y$	0.100/0.068 = 1.47	0.083/0.0305 = 2.72
Case 2a: $X \rightarrow \uptheta$	0.189/0.122 = 1.55	0.094/0.033 = 2.82
Case 2b: $X \rightarrow$ sum ($Y_{\textrm{1}}$ to $Y_{\textrm{4}})$	0.271/0.175 = 1.55	0.207/0.077 = 2.69

TAUF = theoretical augmented uncertainty factor, which equals *q* in Eq. (2). SAUF = simulation-based augmented uncertainty factor, $\hat{q}$. For both TAUF and SAUF, dispersion $1 /m= 0.0064$.

### Adventitious Error and Heterogeneity in Meta-Analysis

When an effect is estimated across several studies in a meta-analysis, either a fixed-effect model or a random-effect model is used. The former model specifies a single effect size for all studies. The latter model allows for effects to vary across studies. In the random-effect model, the standard error of the estimated summary effect (the mean or weighted mean) across studies consists of two components: one that is attributed to sampling variation and another that stems from true variation of the effect across studies (i.e., heterogeneity of the effects). The purpose of this section is to answer Question 2 of Part 2: “How might one interpret the heterogeneity of effects such as correlations in meta-analysis from the perspective of adventitious error?”

To discuss a possible link between adventitious error and the heterogeneity of effects, we use the Cohen’s *d* scale. Let us denote the first component of the standard error variance as $\text {SE}_\textrm{samp}^{2}$, with a subscript “samp” to indicate that its source is sampling variation. $\text {SE}_\textrm{samp}^{2}$ tends to decrease with increasing total number of observations, which is $N\times S$ for *S* studies with equal sample size *N*; $\text {SE}_\textrm{samp}^{2}={var} / {NS}$, where *var* is the within-study variance about the study-specific effect. The second component is $\text {SE}_\textrm{het}^{2}$, with subscript “het” to indicate that it is based on between-study heterogeneity. $\text {SE}_\textrm{het}^{2}$ tends to decrease as the number of studies, *S*, increases: $\text {SE}_\textrm{het}^{2}={\tau ^{2}} / S$, where $\tau ^{2}$ is the variance of the study-specific effects. Using Cohen’s *d* scale for meta-analysis, let $\tau ^{2}$ be in the standard scale. The SE of a meta-analyzed estimate of *d*, $\text {SE}(d_\textrm{met})$, is the square root of the sum of the two components: $\text {SE}\left( d_\textrm{met} \right) =\sqrt{\text {SE}_\textrm{samp}^{2}+SE_\textrm{het}^{2}}$. Unless the number of studies is small, a random-effect approach provides a useful starting point (e.g., Dettori et al., 2022; Tufanaru et al., 2015).

The specific question is to what extent does adventitious error, if it occurs, contribute to the heterogeneity of effects, in terms of standardized $\tau$(i.e., the SD of the effects absent of sampling variation)? We focus our query on correlations^1^ as effects, using Cohen’s *d* as an effect-size measure, in line with common practice. The equation that expresses a correlation in the scale of Cohen’s *d* is:

3 $$\begin{aligned} d={2r} / {(1-r^{2})}, \end{aligned}$$

with *r* to denote the correlation. In the same metric, the corresponding SE for a correlation observed in one study is:

4 $$\begin{aligned} \text {SE}\left( d \right) =2 / \sqrt{\left( N-1\right) (1-r^{2})}, \end{aligned}$$

based on Borenstein et al. (2009). The question is how much larger would this$$ SE$$ be in the presence of adventitious error at the benchmark rate ($$1 / m=0.0064)$$. In what follows, we compare the augmented SE with the SE from Eq. (4) to estimate standardized $$\tau $$ as a measure of heterogeneity to quantify the effect of adventitious error in the context of meta-analysis.

For a correlation of .49, for example between the MVs in Sect. 2.1, $d=1.290$, which is a very large effect. Following Eq. (4), the corresponding SE associated with the correlation for $N = 200$ is 0.163 on the Cohen’s *d* scale. Note that this value is for just one study. The TAUF for an adventitious error with the benchmark rate and $N=200$ is 1.508, so that the SE for one study is augmented to $\text {SE}\times q= 0.163 \times 1.508 = 0.246$. In terms of variance, the corresponding values are $0.163^{\textrm{2}} =0.027$ (non-augmented) and 0.246$^{\textrm{2}} = 0.061$ (augmented). For one study, $S=1$, standardized $\tau ^{2}$ would be 0.061 $- 0.027 = 0.034$, and standardized $\tau$ would be 0.184. According to overviews in the literature (De Boeck and Jeon, 2018; Stanley et al., 2018; van Erp et al., 2017), this estimate of $\tau$ reflects a moderate amount of heterogeneity. In comparison, the corresponding standardized $\tau$ for a correlation of .25 is 0.165, which is not much different from the 0.184 for a correlation of .49. Based on overviews of meta-analysis heterogeneity values, the above values are well within the expected range.

It seems that at least for rather typical correlations and sample sizes, one can expect a non-ignorable level of between-study heterogeneity in the presence of adventitious error. However, we do not claim that heterogeneity of effects is necessarily caused by adventitious error. For example, fixed effects of moderator variables can also lead to heterogeneity. We only wanted to illustrate how a benchmark level of adventitious error is in line with moderate levels of effect-size heterogeneity.

On the condition that a multilevel model for covariance matrices with a dispersion parameter for the IW distribution could be estimated, increasing the number of studies may lead to (a) a more precise estimate of the variation (but not to its reduction) and (b) smaller SEs of the parameter estimates of the theoretical population covariance matrix. Unfortunately, as far as we know, the condition of an estimable model with adventitious error is not met. In contrast, the two effects of increasing the number of studies described in (a) and (b) are available in practice for meta-analysis: more precise estimates of heterogeneity and the overall effect.

### Adventitious Error, Power, and Sample Size

In this section, we outline the implications of adventitious error for power and sample size, using simple regression (like case 1 of Sect. 2.2) as an example. The purpose of this section is to answer Question 3 of Part 2: “What are the consequences of adventitious error for statistical power when testing the significance of correlations and regression slopes in simple regression?”

In particular, we will discuss the case of correlations between two MVs.^2^ If the variables have a variance of one or have equal variance (as in Sect. 2.1), the regression slope *b* equals the correlation. For calculations, we use the Fisher *z* transformation, *z*(*r*). When adventitious error is ignored, the traditional $\text {SE}=\sqrt{1 / {(N-3)}}$. When it is included, Eq. (2) implies that the augmented $\text {SE}=\sqrt{\left( \frac{N-1}{N-3} \right) \frac{1}{m}+\frac{1}{N-3}}$, where 1/*m* is the dispersion of adventitious error. As *N* becomes very large, the traditional SE approaches zero, whereas the augmented SE approaches $\sqrt{1 / m}$.

Adventitious error does not merely increase the SE; it does so in a way that is impervious to increases in sample size. Note that for our benchmark dispersion values of 0.0064 and 0.0025, $\sqrt{1 / m}$ equals 0.08 and 0.05, respectively. The asymptotes for the SE (when sample size is infinitely increased) imply wide confidence intervals. For an observed correlation of .25, for example, the 95% confidence interval cannot be narrower than [.098, .390] when dispersion $= 0.0064$ or narrower than [.156, .339] when dispersion $= 0.0025$.

**Fig. 3:**
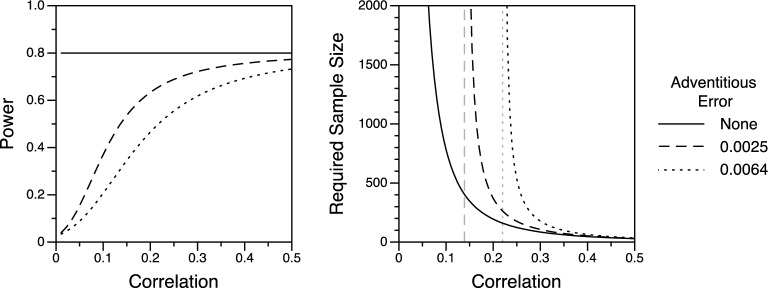
The effects of adventitious error on power and required sample sizes for detecting significant correlations. Left panel: For each correlation, the sample size required for power $=.80$ with no adventitious error was also used for the two nonzero levels of adventitious error, yielding lower power. Right panel: Sample sizes required for power $=.80$ at three levels of adventitious error. For the two nonzero levels of adventitious error, the vertical asymptotes in gray indicate correlations below which it is impossible to achieve power $=.80$.

It is easy to show the consequences for statistical power based on the augmented SE and the SE asymptotes as just discussed. Consider power when $\alpha =.05$ and the alternative hypothesis is that *r* has some positive value, such as .25. For simplicity, we ignore the chance of obtaining a significant negative result; in effect, we assess one-tailed power with $\alpha =.025$. For $r =.01$ to .50, we computed the sample size required for power $=.80$ (“a convention used for general use”; Cohen, 1992, p. 156) in the usual way, ignoring adventitious error. For example, the required *N* is 124 when $r =.25$ and 47 when $r =.40$. For each of these (*r*, *N*) pairs, we then computed power using the augmented SE corresponding to the inclusion of adventitious error (we used unrounded values for *N*). For dispersion $= 0.0064$, power $=.55$ when $r =.25$ and $N = 124$, and power $=.69$ when $r =.40$ and $N = 47$. The full curves for dispersion $= 0.0025$ and 0.0064 are shown in the left panel of Fig. 3. In the figure, power is always .80 when adventitious error is ignored (when dispersion $= 0$) because the *N*s were selected to yield that level of power for every correlation. Using those same *N*s, power is substantially lower when adventitious error is considered, especially when correlations are small.

How much larger must *N* be, relative to the usual value, to attain power $=.80$ for a given value of *r*? Let $N_{\textrm{1}}$ and $N_{\textrm{2}}$ be the required sample sizes for a planned study when adventitious error is ignored and considered, respectively. The goal is to choose $N_{\textrm{2}}$ such that the augmented SE (considering adventitious error) equals the traditional SE (ignoring adventitious error). From the expressions above, we have $\sqrt{\frac{1}{N_{1}-3}} =\sqrt{\left( \frac{N_{2}-1}{N_{2}-3} \right) \frac{1}{m}+\frac{1}{N_{2}-3}}$. Solving for the larger sample size yields

5 $$\begin{aligned} N_{2}=\frac{N_{1}-\left( N_{1}-3 \right) \left( 1 / m \right) }{1-\left( N_{1}-3 \right) \left( 1 / m \right) }. \end{aligned}$$

One can calculate the required $N_{\textrm{1}}$ in the usual manner and then scale it up to get the required $N_{\textrm{2}}$. For dispersion $1 / m = 0.0064$, the required *N* increases from 124 to 533 for $r =.25$ and from 47 to 65 for $r =.40$; the increase is much more dramatic for the smaller correlation. The curves for dispersion $= 0.0025$ and 0.0064 are shown in the right panel of Fig. 3, along with the curve for the required sample size when adventitious error is ignored (dispersion = 0).

In the figure, the three curves for the required sample size have different vertical asymptotes. In the absence of adventitious error, the asymptote is $r = 0$. One can obtain power $=.80$ for arbitrarily small hypothesized nonzero correlations if one collects a large enough sample (e.g., an *N* of nearly 20,000, which is off the chart, for $r =.02$). However, in the presence of adventitious error, the asymptote can be noticeably larger than $r = 0$. Recall that in this condition, the augmented SE approaches $\sqrt{1/m}$ as the sample size increases without bound. To achieve power of .80, (the Fisher *z* of) the alternative hypothesis value of *r* must be about $0.84\times \text {SE}$ greater than the critical value, which is $1.96\times \text {SE}$. In other words, the alternative hypothesis value of *z*(*r*) must be at least $\left( 0.84+1.96 \right) \times SE$, where the *SE* in question is now the augmented version ($\text {SE}\times q)$. For dispersion $= 0.0064$, the threshold value for *z*(*r*) is $2.80\times \sqrt{0.0064} =0.224$, or $r =.220$. For correlations at or below this threshold, it is impossible to achieve 80% power in a single study, regardless of its sample size. For dispersion $= 0.0025$, the threshold for *r* is .139. (Below these thresholds, the above equation for $N_{\textrm{2}}$ does not hold because the traditional and augmented SEs cannot be equal for positive values of $N_{\textrm{2}}$ when $\frac{1}{{(N}_{1}-3)}<\frac{1}{m}$.)

Not only is power reduced and bounded in the presence of adventitious error because of the augmented SE, there also are consequences for whether observed correlations can be significant. The null hypothesis cannot be rejected in a single study of any size if (the Fisher *z* of) the observed correlation is below 1.96 times the smallest possible augmented *SE*. For example, correlations below .157 (for dispersion $= 0.0064$) or below .098 (for dispersion $= 0.0025$) cannot lead to a rejection of the null hypothesis. If adventitious error behaves like heterogeneity, as suggested in the previous section, it would require multiple studies to reduce the uncertainty about the summary effect. Compared with the traditional assertion that all null hypotheses are false, adventitious error implies a more nuanced view. Due to adventitious error, theoretical correlations of zero may be distorted to nonzero correlations. Choosing a dispersion value for adventitious error could be a way to set boundary values to prevent false rejections of the null hypothesis.

The implications of adventitious error for power, sample size, and the values below which parameter estimates cannot lead to a rejection of the null hypothesis parallel those of effect-size heterogeneity in meta-analysis (Kenny and Judd, 2019; McShane and Böckenholt, 2014; Pawel and Held, 2020; Pek et al., 2024), which is not surprising given the discussion in Sect. 2.2. Here, we highlight one implication that has thus far received insufficient attention: Although increasing the sample size beyond that recommended by traditional power analysis is essential for maintaining power when the hypothesized effect is modest (e.g., $r =.30$), the same strategy may be futile when the hypothesized effect is small (e.g., $r =.10$) because no sample size will suffice. For small observed values, it may be impossible to reject the null hypothesis in a single study if heterogeneity or adventitious error is considered.

## Adventitious Error and Measurement Uncertainty

### Measurement Error and Adventitious Error

Measurement error is the deviation of a measurement outcome from the expected value for hypothetical independent repeated measurement outcomes for the same individual under the same standard condition. The expected value is taken over a propensity distribution of independent repeated outcomes using measurement tool *g* for an individual in the standard condition. The expected value is also called the true value, denoted here as $t_{gi}$, with *i* as an index for individuals. The deviation of a measurement outcome $X_{gi}$ from the expected value $t_{gi}$ is the measurement error for an individual, $\varepsilon _{gi}$, so that $X_{gi}=t_{gi}+\varepsilon _{gi}$. The variance of $\varepsilon _{gi}$ per individual (across the propensity distribution for individual *i*), $\sigma _{\varepsilon i}^{2}$, cannot be directly estimated, but the average error variance across individuals (the average across individuals of the within-individual $\sigma _{\varepsilon i}^{2})$ can be estimated through the variance of measurement error as a random variable across individuals (Lord & Novick, 1968, p. 35), denoted here as $\sigma _{e}^{2}$. An estimate of $\sigma _{e}^{2}$ is often taken as an estimate of $\sigma _{\varepsilon i}^{2}$, and $\sigma _{e}^{2}$ is used as the standard error of measurement (S.E.M.), an inverse index of measurement precision. Thus, a between-individual parameter ($\sigma _{e}^{2})$ is used as a statistical proxy for a within-individual parameter ($\sigma _{\varepsilon i}^{2})$.

Multiple items (manifest variables, MVs) are commonly used for the measurement of constructs. For a summed score based on a set of items, the S.E.M. can be estimated, for example, with coefficient alpha or McDonald’s omega or some other reliability coefficient depending on assumptions about the items (e.g., tau equivalence for the alpha coefficient). Alternatively, factor scores can be used as surrogates for the construct, in which case the variance of regression factor scores is taken as a reliability coefficient of the scores, which can in turn be used to estimate the S.E.M.

Lord and Novick (1968) interpreted measurement error as “a disturbance that is due to a composite of a multitude of factors not controlled in the measurement procedure” (p. 38). That measurement error is formulated as random can be understood as a way to deal with unspecified complexities about factors with an effect on the observed measurement outcome. The question we want to address in this third part is how to consider adventitious error in relation to measurement error. Like measurement error, $\sigma ^2_{\varepsilon _i}$, can we obtain an estimate of the within-individual variance (and standard deviation, SD) due to adventitious error? If so, the SD due to adventitious error would give us an indication of the impact of adventitious error on the uncertainty of measurement outcomes for an individual using a given measurement tool, in a similar way as the S.E.M. does for measurement error.

However, unlike measurement error, adventitious error is associated with the variances and covariances of MVs in ways that reflect the implementation of a specific study and is thus irrelevant to the theoretical structure of the MVs. For example, when a set of MVs is used to measure a construct, adventitious error generates deviations from the theoretical relations, and those deviations are conceptually irrelevant to the construct. An alternative view is that the nature of the construct itself is not fixed, as discussed by De Boeck et al. (2023), but here we stay with the common view that the nature of a construct is fixed and reflected in fixed relations of the factors (latent variables) with the MVs, except that this common view ignores the distinction between the theoretical population and the operational population. It is self-evident that measurement error has implications for the variation and uncertainty of measurement outcomes. The consequences for measurement outcomes due to adventitious error are less obvious, which is why a simulation study may help.

### Purpose of the Simulation Study

The purpose of the simulation study is to assess the variation in measurement outcomes (e.g., summed scores and factor scores) that is specifically induced by imposing adventitious error on data from a one-dimensional CSM. Our simulation allows us to isolate adventitious error from variation based on measurement error (i.e., S.E.M.) and from the sampling variation that normally arises in the generation of multivariate data. This separation can be realized by reformulating adventitious error as a linear transformation of a set of variables and by taking advantage of the internal algorithmic process when using the mvrnorm( ) function (see Song, 2021, for an explication of the algorithm). In this process, the linear transformation that adds adventitious error is at the last stage, as explained in Sect. 3.3. For a more accurate estimate of the impact of adventitious error on the variation of measurement outcomes for the same individuals within a single sample, the simulation is repeated for different simulated samples of individuals. As mentioned, we focus on composite scores (summed scores and factor scores) because most measurement outcomes are composite scores. Given that composite scores change when the set of variables involved in the composites are linearly transformed due to a change of the covariance matrix of the variables, we expect that the composite scores will be affected by adventitious error.

### Design and Algorithm

The design of the simulation study is that 1000 data sets are generated for each of 10 different independent simulation samples, each with 1000 individuals and four MVs. The 1000 data sets *within *a simulation sample differ only with respect to adventitious error, and not in any other respect such as measurement error of sampling variation. *Between *simulation samples, the variability is a reflection of sampling variability. The total number of data sets to be generated is 10 $\times$ 1000.

As mentioned, the measurement outcomes to be considered for these data sets (1000 data sets for each of the 10 samples) are simple summed scores and one-factor model factor scores per individual based on the four MVs. Per individual within a sample, there are 1000 summed scores and 1000 factor scores. These composite measurement outcomes per individual *within *a simulation sample vary depending on the data set, and this variation is due solely to adventitious error.

For the simulation algorithm, the following steps are repeated for each of the 10 simulation samples, with $k=1, .., K=10$, as an index for the samples.

Step 1. Generate 1000 independent operational covariance matrices $\varvec{\varSigma }_{s_{k}}$ with size $p\times p$, where $p=4$ as the number of MVs, and $s_{k}=1, .., S_{k}$, as an index for the data sets to be generated for sample *k*, based on the following inverse Wishart distribution:

6 $$\begin{aligned} \varvec{\varSigma }_{s_{k}}\vert \varvec{\varOmega }, m \sim IW(m\varvec{\varOmega },m), \end{aligned}$$

where $\varvec{\varOmega }$ is the theoretical covariance matrix, and $m=1/RMSEA^{2}$.

For our simulation study, $m=\textrm{1} / {\mathrm {0.0}\textrm{8}^{\textrm{2}}}$ (0.08 is the reference RMSEA value associated with a benchmark level of adventitious error); and

$$\begin{aligned} \varvec{\varOmega } =\left[ {\begin{array}{cccc} 1 &{} 0.49 &{} 0.49 &{} 0.49\\ 0.49 &{} 1 &{} 0.49 &{} 0.49\\ 0.49 &{} 0.49 &{} 1 &{} 0.49\\ 0.49 &{} 0.49 &{} 0.49 &{} 1\\ \end{array} } \right] , \end{aligned}$$

corresponding to a one-factor model with loadings of 0.70 and residual variances of 0.51 for all four MVs. There are 10 x 1000 $\varvec{\varSigma }_{s_{k}}$ matrices. The variation between these covariance matrices reflects adventitious error. Note that the $\varvec{\varSigma }_{s_{k}}$ matrices do not depend on *k*; they are just being used for sample *k*.

Step 2. Generate a random seed $h_{k}$ for sample *k*, to be used in step 3.

Step 3. Generate 1000 $N\times p$ data sets ${\varvec{X}}_{s_{k}}$ for sample *k*, where $N=1000$, based on the following multivariate normal distribution:

7 $$\begin{aligned} {\varvec{X}}_{s_{k}}\vert h_{k},\varvec{\varSigma }_{s_{k}},\varvec{\mu }\sim MVR(\varvec{\varSigma }_{s_{k}}{,{\varvec{\mu }} }), \end{aligned}$$

where $\varvec{\mu } ={\textbf {0}}$, a vector with length *p*.

For each value of $s_{k}$, the generation of ${\varvec{X}}_{s_{k}}$ consists of two substeps:

Step 3a. Generate an orthonormal preliminary data set ${\varvec{Z}}_{k}$ with size $N\times p$ using random seed $h_{k}$ and based on the following multivariate normal distribution:

8 $$\begin{aligned} {\varvec{Z}}_{k}\vert h_{k}{, {\varvec{I}}, {\varvec{\mu }} }\sim MVR(\varvec{\mu },{\varvec{I}}), \end{aligned}$$

where ${\varvec{I}}$ is a $p\times p$ identity matrix. The random seed $h_{k}$ ensures that the individuals from the 1000 data sets within each *k*th sample are the same.

Step 3b. Linearly transform ${\varvec{Z}}_{k}$ into ${\varvec{X}}_{s_{k}}$ using $\varvec{\varSigma }_{s_{k}}$ (based on an eigen-decomposition of $\varvec{\varSigma }_{s_{k}})$, resulting in 1000 data matrices ${\varvec{X}}_{s_{k}}$ for sample *k*. Thus, the variation of ${\varvec{X}}_{s_{k}}$ between the 1000 data sets within each *k*th sample reflects adventitious error.

Through executing the three steps for each of the 10 samples, we obtain 10 $\times$ 1000 data sets. The 1000 data sets within each sample are dependent as linear transformations of the same preliminary data set. They also are linear transformations of each other, induced by the variability of adventitious error.

### Measurement Outcomes

For each sample’s 1000 data matrices ${\varvec{X}}_{s_{k}}$, a one-factor model is fit to the data using lavaan with ML estimation, resulting in estimated regression factor scores (Thurstone, 1935). In addition, the summed scores (sums of the four MVs) are determined per individual. This yields 1000 factor scores and 1000 summed scores per individual in each of the 10 samples, one factor score and one summed score for each of the 1000 data sets in sample *k*. These factor scores and summed scores are standardized within each data set, so that all measurement outcomes have the same scale and can be compared. The downsides of this standardization are that (a) the variation across the data sets within a simulation sample is reduced because the variation of SDs due to adventitious error is eliminated and (b) standardization has scale effects.

Per individual (which is the same across the $S = 1000$ data sets per sample because the random seed is the same for each *k*), the SD across 1000 data sets is calculated for the factor scores and for the summed scores. These SDs are a quantification of the measurement outcome variation due to adventitious error.

Finally, the SDs per individual are averaged across all individuals from the same *k*th simulation sample, for factor scores and for sum scores. In this way, each sample provides an independent estimate of the average within-individual SD for factor scores and for summed scores.

### Results and Discussion

The results are reported in Table 2 and can be summarized as follows:

The findings can be generalized across the 10 samples. The within-individual SD for factor scores of the 10 samples is on average 0.084, roughly 8.4% of the between-individual SD of factor scores (which is 1 because of the standardization). The estimated within-individual SD would lead to a 95% confidence range with a width of roughly 0.33 on the standard scale (the difference between 0.084 $\times 1.96$ and $-$ 0.084 $\times$ 1.96). The within-individual SD for summed scores is approximately 0.035, roughly 3.5% of the between-row SD (which is 1). This would lead to a 95% confidence range with a width of 0.14 on the standard scale (the difference between $0.035 \times 1.96$ and $-0.035 \times$1.96). These CIs represent variation due to adventitious error.

The within-individual variation across data sets within samples (i.e., solely due to adventitious error) is smaller for sum scores than for factor scores, although the variation is measured on the same scale. The difference can be explained by the fact that the MV weights for factor scores (but not for summed scores) vary with the operational covariance matrix. The factor score coefficients (i.e., the weights) to estimate the factor scores depend on $\varvec{\varSigma }_{sk}$, but the weights for summed scores do not. As explained earlier, the differences between the data matrices for the same sample *k* are not affected by sampling variation because all data sets of the same sample are linear transformations of each other, for factor scores and summed scores alike, so that sampling variation cannot explain the differences in SDs for the two types of composite scores.

For factor scores and summed scores, the within-individual variation due to adventitious error may seem small compared with the variation due to measurement error. For purposes of comparison, we use a reliability coefficient of .90, a value that may be considered high for research purposes, given Cronbach’s (1951) appraisal of 0.70 as fairly large and given that Nunnally (1978) and the APA Dictionary of Psychology (Vandenbos, 2015) recommend 0.80. For a standardized scale, the corresponding S.E.M. is 0.316, and the width of the 95% confidence range is 1.24 (the difference between $0.316 \times 1.96$ and $-\,0.316 \times 1.96)$, which is more than 100% of the SD of standardized between-individual differences. These high values may be surprising, but what looks good in terms of reliability coefficients is not so good in terms of S.E.M. This explains why Nunnally (1978) recommends a higher reliability coefficient for decisions based on individual test scores than for research.

There are three possible reasons for the small variation induced by adventitious error:

A. First, the adventitious error rate corresponds to an acceptable GOF for CSM-based measurement models. Therefore, the small within-individual SDs are not surprising. Our result can be interpreted as corroborating evidence for considering an RMSEA value of 0.08 as acceptable.

B. Second, the factor scores and summed scores in our simulation rely on four MVs with an average inter-MV correlation of .49, so the composite scores should be relatively stable. Recall that in our simulation study in the introductory section, the mean absolute difference between operational correlation matrices due to adventitious error was only 0.079. When the loadings of the four MVs of the one factor are reduced to 0.50 so that the theoretical correlations among the MSs are .25, and a simulation study is conducted with these new values, we find that the average SDs of the standardized factor scores and the summed scores increase by a factor of 2.5.

C. Finally, the within-individual SDs of factor scores and summed scores resulting solely from adventitious error are rather small because they stem from linear transformations of the same sample-specific preliminary data set ${\varvec{Z}}_{k}$.

**Table 2:** Simulation results for within-individual variation of composite measurement outcomes based only on adventitious error.

Simulation sample	Mean within-individual SD of standardized regression factor scores	Mean within-individual SD of standardized summed scores
1	0.096	0.034
2	0.080	0.034
3	0.073	0.035
4	0.082	0.034
5	0.087	0.034
6	0.092	0.036
7	0.078	0.035
8	0.087	0.034
9	0.071	0.035
10	0.094	0.035
Grand mean	0.084	0.035

Means of within-individual SDs were computed across 1000 individuals per sample. The within-individual SDs were based on 1000 randomly generated covariance matrices designed to represent adventitious error, using the benchmark adventitious error rate (0.0064) and in the absence of measurement error and sampling error.

## General Discussion

In Part 2, we have illustrated the consequences of adventitious error for the SE of a *relationship between variables*. In Part 3, we have focused on *composite measurement outcomes* that are (weighted or unweighted) sums of four MVs. The consequences of adventitious error seem larger for the uncertainty of relations than for the uncertainty of composite measurement outcomes. A possible reason is that the results of Part 3 concern aggregate level statistics (weighted and unweighted summed scores), whereas the results of Part 2 concern relation statistics (i.e., relations between pairs of variables).

Two features of adventitious error should be emphasized for a more nuanced interpretation of the results. First, adventitious error implies random disturbances of the operational covariance matrix ($\varvec{\varSigma }_{s})$ from the theoretical covariance matrix ($\varvec{\varOmega })$. Therefore, adventitious error cannot lead to systematic bias (i.e., an expected nonzero difference), but it could lead to a random discrepancy with true parameter values in a single study or a small set of studies due to the random (non-systematic) distortion it introduces. Second, adventitious error is unrelated to random sampling of MVs that reflect a construct. Random sampling of MVs (i.e., items rather than participants) can have other and possibly more drastic effects on the within-individual SD of measurement outcomes compared with adventitious error because adventitious error can be reduced to linear transformations of the same MVs, whereas sampling MVs implies that different sets of MVs are used.

To summarize, the benchmark adventitious error rate (based on RMSEA $= 0.08$ for CSMs) has serious consequences for the uncertainty regarding relations between variables and seemingly less serious consequences for the uncertainty of measurement outcomes. However, this conclusion depends on the metric one wants to use to appraise the consequences. If adventitious error is present, one may also expect heterogeneity of effects in meta-analytic studies and (if adventitious error is ignored) an overestimation of power, especially for smaller effect sizes.

A key limitation of our contribution is that there is no empirical proof of adventitious error. Instead, we consider adventitious error as a framework, based on the assumption that all models are wrong (i.e., have approximate GOF). The framework can be useful for interpreting well-established phenomena such as heterogeneity of effects, overestimated power, and variability in research findings generally. That these phenomena follow from the same hypothetical principle of adventitious error is not a proof of that principle, but rather an illustration that the principle provides a parsimonious description of seemingly unrelated regularities.

Another limitation is that our simulations are far from comprehensive. We have mainly used one adventitious error rate (i.e., 0.0064, corresponding to an RMSEA of 0.08 for CSMs), a covariance matrix associated with a one-dimensional factor model (with rather high MV correlations), and only a couple levels of sample size. More extensive simulation studies would be desirable.

A final limitation is that, unfortunately, a multilevel model with a dispersion parameter for covariance matrices is at present not within reach. If it were, one could use data from a rather large number of studies to estimate the adventitious error dispersion as a parameter of that multilevel model. Available multilevel factor models are based on the assumption of homogeneous covariance matrices, except for possible differences in the variances of the factors (Muthén, 1994; Stapleton et al., 2016). Other multilevel approaches focus either on variation per cell of the covariance matrix (Cheung and Chan, 2005) or on a specific effect in a structural equation model (Preacher et al., 2010), without considering the random variation of the whole covariance matrix. Even so, a multilevel model with the dispersion of adventitious error as a parameter would still not solve the problems of adventitious error associated with (a) single studies or (b) studies with single variables or data analyses without covariance structure modeling. Even with such a multilevel model available, one cannot rely on estimated adventitious error rates in those situations. As a final consideration, although we have used benchmark values for the adventitious error rate to illustrate the consequences for inferences regarding relations between variables and for the uncertainty of composite measurement outcomes, we refrain from recommending benchmark values for actual inferences in practice.

We conclude that a possible cause of approximate fit, in the form of adventitious error, has consequences for the uncertainty on relations between variables and on composite measurement outcomes. The custom of accepting models with approximate fit may have further consequences beyond having a useful model. Whereas approximate models can certainly be useful, their approximate fit also suggests that there is more uncertainty in research findings and in measurement than we commonly suppose there is.

## Footnotes

1 Because correlations are bounded, the conclusions are only approximate.

2 Because correlations are bounded, unlike covariances, the *q* factor might not apply strictly to correlations. Applying the Fisher *z* transformation to correlations does not solve this potential problem. Although our results are only approximate, they remain consistent with our main conclusions. .
